# CDKN2B is critical for verapamil-mediated reversal of doxorubicin resistance in hepatocellular carcinoma

**DOI:** 10.18632/oncotarget.22123

**Published:** 2017-10-26

**Authors:** Tengyue Zhang, Kelong Ma, Jin Huang, Shitang Wang, Yabei Liu, Gaofei Fan, Miao Liu, Guangshan Yang, Cheng Wang, Pingsheng Fan

**Affiliations:** ^1^ School of Clinical Medicine, Shan Dong University, Jinan 250100, China; ^2^ The Cancer Hospital of Anhui Province, Provincial Hospital of Anhui Medical University, Hefei 230032, China; ^3^ Clinical College of Integrated Traditional Chinese and Western Medicine, Anhui University of Chinese Medicine, Hefei 230032, China; ^4^ Department of General Surgery, Provincial Hospital of Anhui Medical University, Hefei 230032, China

**Keywords:** hepatocellular carcinoma (HCC), verapamil (VER), doxorubicin (ADM), CDKN2B, chemotherapy resistance

## Abstract

In this study, we explored the function and mechanism of CDKN2B genes in verapamil (VER)-induced reversal of resistance to doxorubicin (ADM) chemotherapy in hepatocellular carcinoma (HCC). We examined 4 HCC cell lines and found that the expression levels of CDKN2B genes correlated with the level of apoptosis induced by ADM+VER. Overexpression of CDKN2B genes promoted apoptosis in cells treated with VER+ADM. CDKN2B knockdown using siRNA weakened the effect of ADM+VER, indicating that ADM+VER promotes HCC cell apoptosis and that CDKN2B genes participate in VER-mediated promotion in tumor cell apoptosis. Future research will further explore the functional mechanism, and the associated signal transduction pathways via which CDKN2B affects HCC drug resistance.

## INTRODUCTION

HCC is a common substantive tumor in China and the second most malignant tumor in terms of fatality rate and threat to public health [1]. Most patients are diagnosed in the middle and terminal stages after the optimal surgical opportunity has passed because of the long latency and quick development of HCC. Transcatheter arterial chemoembolization (TACE) is an important therapy for these middle and terminal stage HCC patients [2]. Chemotherapy and targeted drugs are the main means to prolong the survival time of HCC patients, but their therapeutic effect is limited by drug resistance. Only approximately 50% of current HCC responds to standard chemotherapy and sorafenib, which is the most effective targeted drug. Even among these responders, the survival time is only prolonged by 3 months, based on the systematic analysis of multiple random clinical control test results [3, 4].

Verapamil (VER) can reverse the chemotherapy drug resistance of multiple types of tumor cell [5]. An *in vitro* study reported that 6.0–10.0 μmol/L VER concentration, which is higher than the safe vein concentration (1.0–2.0μmoL/L), can effectively reverse tumor drug resistance. Exceeding the safe vein concentration of VER causes sinus bradycardia, atrioventricular block, and other serious toxic side effects [6], thus restricting its clinical application as a reversal agent of tumor multidrug resistance. In this study, we sought to explore the effect of TACE and altered expression of CDKN2B in order to increase the effectiveness of VER reversal of chemotherapy resistance.

## RESULTS

### Evaluation of VER reversal of resistance to three chemotherapeutic agents in four HCC cell lines

We evaluated the anticancer activity of oxaliplatin (L-OHP), doxorubicin (ADM), and 5-fluorouracil (5-FU) against QGY-7703, HepG2, SMMC-7721, and BEL-7402 cell lines in the absence or presence of VER (4.91μg/ml). The results are summarized in Table 1. The IC_50_ values of L-OHP in HepG2 and BEL-7402 were significantly higher than those in SMMC-7721 and QGY-7703. The IC_50_ value of ADM in QGY-7703 was lower than in the other three cell lines. The IC_50_ value of 5-FU in SMMC-7721 was higher than in the other three cell lines. After VER was added, the IC_50_ values of L-OHP, ADM, and 5-FU in the four cell lines (SMMC-7721, BEL-7402, HepG2, and QGY-7703) declined to different extents, indicating that VER increases sensitivity of the three aforementioned chemotherapeutics to different extents.

**Table 1:** Cytotoxic activity of the examined drugs against QGY-7703, HepG2, SMMC-7721 and BEL-7402 cells^a^

Drug	IC_50_ (μg/ml)^b^			
	QGY-7703	HepG2	SMMC-7721	BEL-7402
L-OHP	53.52 ± 3.64	227.01 ± 18.64	11.98 ± 0.98	330.30 ± 20.87
L-OHP+ VER (4.91μ g/ml)	6.50 ± 0.86	38.09 ± 5.62	10.48 ± 1.42	93.69 ± 9.64
ADM	0.81 ± 0.03	8.40 ± 1.02	5.71 ± 0.13	11.39 ± 0.27
ADM+VER (4.91μ g/ml)	0.63 ± 0.06	2.93 ± 0.14	3.72 ± 0.24	0.69 ± 0.02
5-FU	97.84 ± 5.98	148.41 ± 14.36	445.85 ±23.14	317.35 ± 18.69
5-FU+VER (4.91μ g/ml)	25.61 ± 2.67	29.54 ± 2.03	48.70 ± 5.05	34.08 ± 2.33

Negative control 0.1%DMSO, no activity.

^a^The data represent the mean of three experiments in triplicate and are expressed as means ± SD; only descriptive statistics were done in the text.

^b^The IC_50_ value was defined as the concentration at which 50% survival of cells was observed.

**Figure 1:**
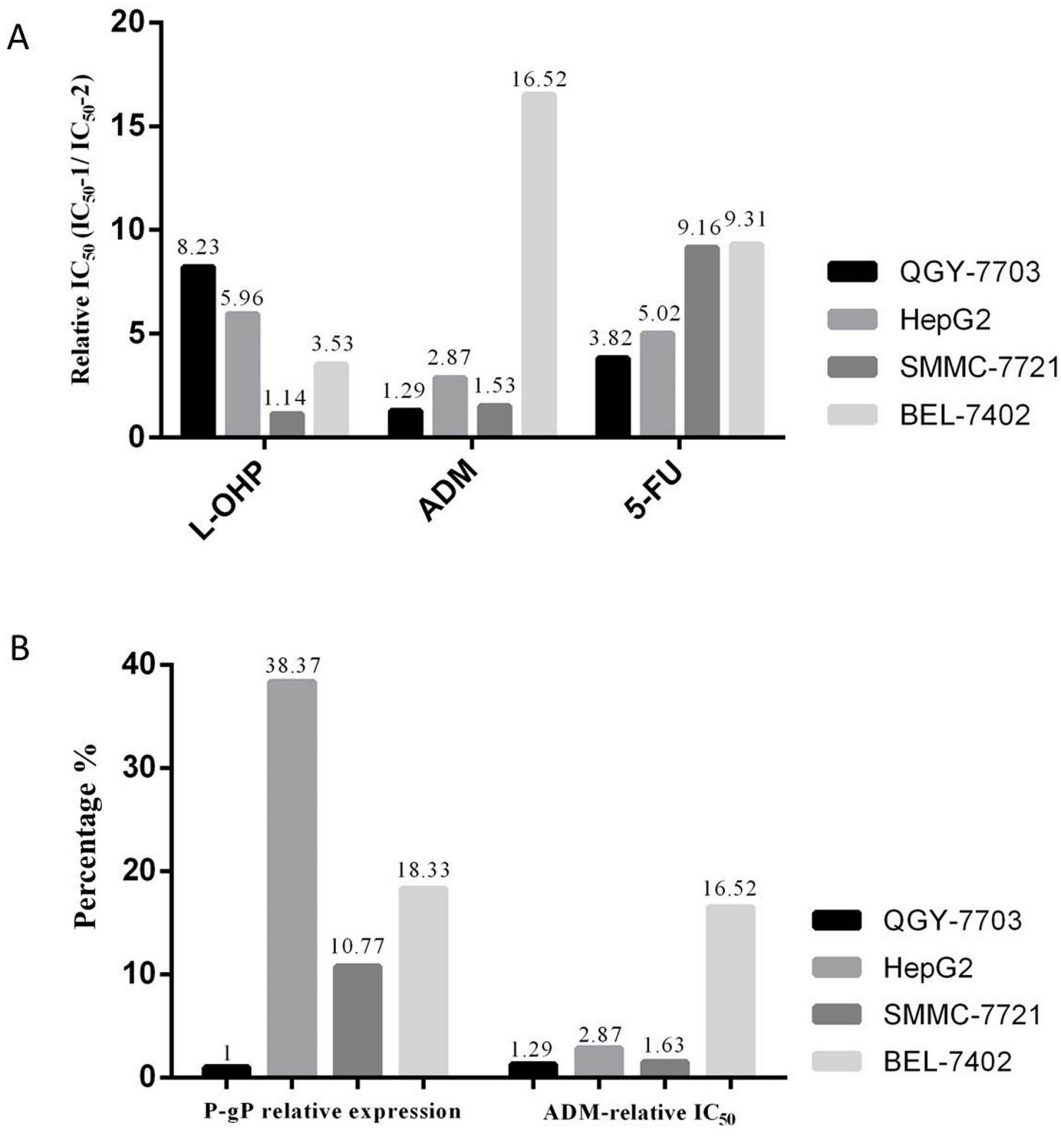
**(A)** Evaluation of VER reversal of ADM resistance, (Relative IC_50_ = IC_50_-1/IC_50_-2). The IC_50_ values of four types of HCC cell lines (SMMC-7721, BEL-7402, HepG2, and QGY-7703), which were treated by chemotherapeutics in the absence (IC_50_-1) or in the presence (IC_50_-2) of VER; **(B)** relationship between expression level of MDR1/P-gP and resistance to VER + ADM (Relative IC_50_).

The continued resistance to VER plus chemotherapy was evaluated using relative IC_50_ =IC_50_-1/IC_50_-2 in this study. IC_50_-1 represents the sensitivities of QGY-7703, HepG2, SMMC-7721, and BEL-7402 to L-OHP, ADM, and 5-FU, respectively, whereas IC_50_-2 represents the sensitivities of QGY-7703, HepG2, SMMC-7721, and BEL-7402 to the VER (4.91 ug/mL)+L-OHP, VER (4.91 ug/mL)+ADM, and VER (4.91 ug/mL)+5-FU, respectively. A relatively high IC_50_ implies high drug resistance. The resistance of BEL-7402 to VER + ADM was strongest (Relative IC_50_=16.52) and significantly different than SMMC-7721 (Relative IC_50_=1.53, Figure 1A).

### Correlation analysis between P-gP expression level and resistance to VER reversal of ADM resistance

We found significant differences in the expression levels of MDR1, a P-gP encoding gene, among the four HCC cell lines using qRT-PCR. The expression levels of MDR1 in SMMC-7721, BEL-7402, and HepG2 cells were higher than in QGY-7703. The expression level of MDR1 was highest in HepG2. BEL-7402 exhibited significantly stronger resistance to VER reversal of ADM resistance (Relative IC_50_=16.52), than the other three cell lines (Figure 1B) based on the IC_50_ value measured. No simple correlation between the expression level of MDR1/P-gP and VER resistance was found.

### Transcriptome sequencing based on Illumina sequencing platform

**Figure 2:**
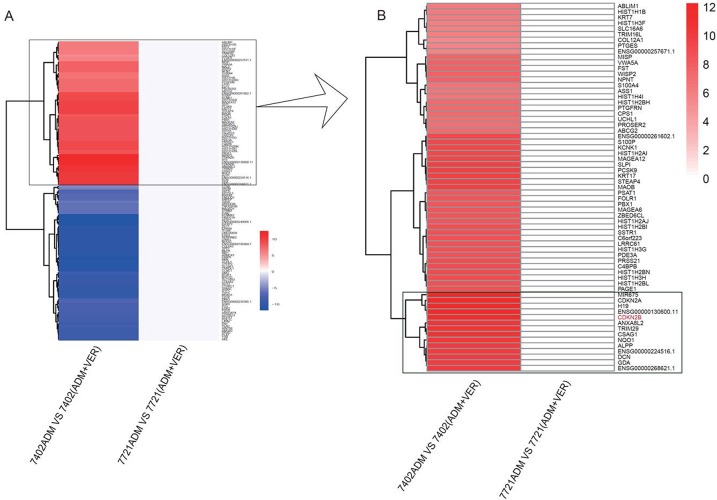
High-flux sequencing thermograph of SMMC-7721 and BEL-7402 cells Entire view **(A)** and view of the significantly different genes **(B)**.

BEL-7402 has a stronger resistance to VER reversal of ADM resistance than SMMC-7721. We used SMMC-7721 and BEL-7402 to screen genes that may mediate resistance to VER reversal of ADM resistance. Using the Illumina 2000 sequencing platform, the raw data was filtered in order to convert the low quality sequence to high quality data (clean reads). We obtained samples of clean reads in line with further analysis requirements. We found a total of 135 genes in BEL-7402 with significant differences in expression (log2 (awei/a)> 1, ^*^p <0.05) that were not altered in SMMC-7721. Of these significantly different genes (Figure 2A), 65 were significantly higher in the BEL-7402 cell line after the use of VER (Figure 2B).

### Real-time quantitative PCR test of candidate gene expression in hepatoma carcinoma cells

**Figure 3:**
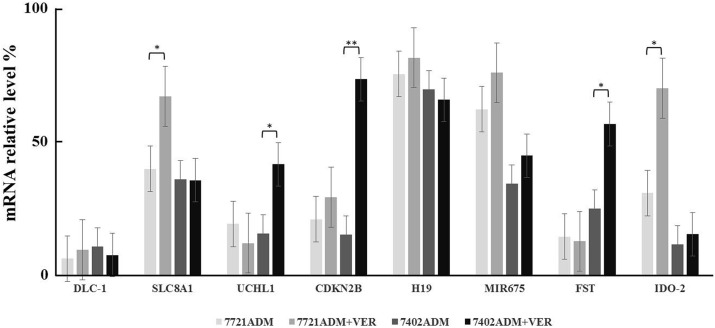
Real-time quantitative PCR test of candidate gene expression in HCC cells Independent experiments were performed throughout the *in vitro* studies in triplicate. ^*^p<0.05, ^**^p<0.01 compared to the ADM group.

Eight upregulated or downregulated genes were selected. Genes related to drug resistance in our literature retrieval were selected as the candidate genes, namely, SLC8A1, DLC-1, FST, CDKN2B, UCHL1, IDO-2, miR-675, H19 (Figure 3). The qRT-PCR showed that the expression of CDKN2B, SLC8A1, UCHL1, FST and IDO-2 was significantly altered after VER treatment. The expression of CDKN2B, UCHL1, and FST genes between the 7721ADM group and 7721ADM + VER group had no significant difference, but were significantly increased in the 7402ADM + VER group over the 7402ADM group (^*^ p <0.05); the CDKN2B gene was the most significantly different (^**^ p <0.01) (Figure 3).

### Western blot test of expression of CDKN2B/P15 protein in hepatoma carcinoma cells

**Figure 4:**
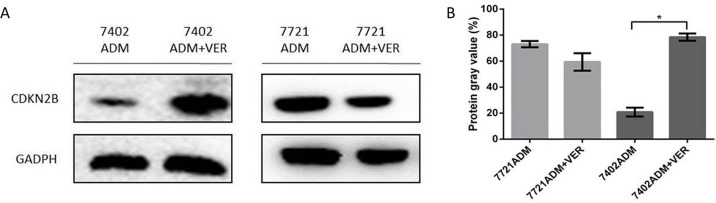
**(A)** Western blot test of expression of CDKN2B/P15 protein in HCC cells. **(B)** The profiles showed the protein gray value (%). Independent experiments were performed throughout the *in vitro* studies in triplicate. ^*^p<0.05 compared to the ADM group.

We analyzed the expression of CDKN2B/P15 protein in HCC cell lines. Figure 4 shows that the expression of CDKN2B/P15 protein was not significantly different between 7721ADM and 7721ADM+VER, but significantly increased in 7402ADM+VER over 7402ADM (^*^p<0.05). The expression of CDKN2B/P15 protein in SMMC-7721 cells was not significantly changed before or after VER was used, but the expression of CDKN2B/P15 protein was significantly increased in BEL-7402 cells.

### Immunohistochemical test of expression of CDKN2B/P15 protein in patients receiving VER+ transcatheter arterial chemoembolization (TACE) therapy

**Figure 5:**
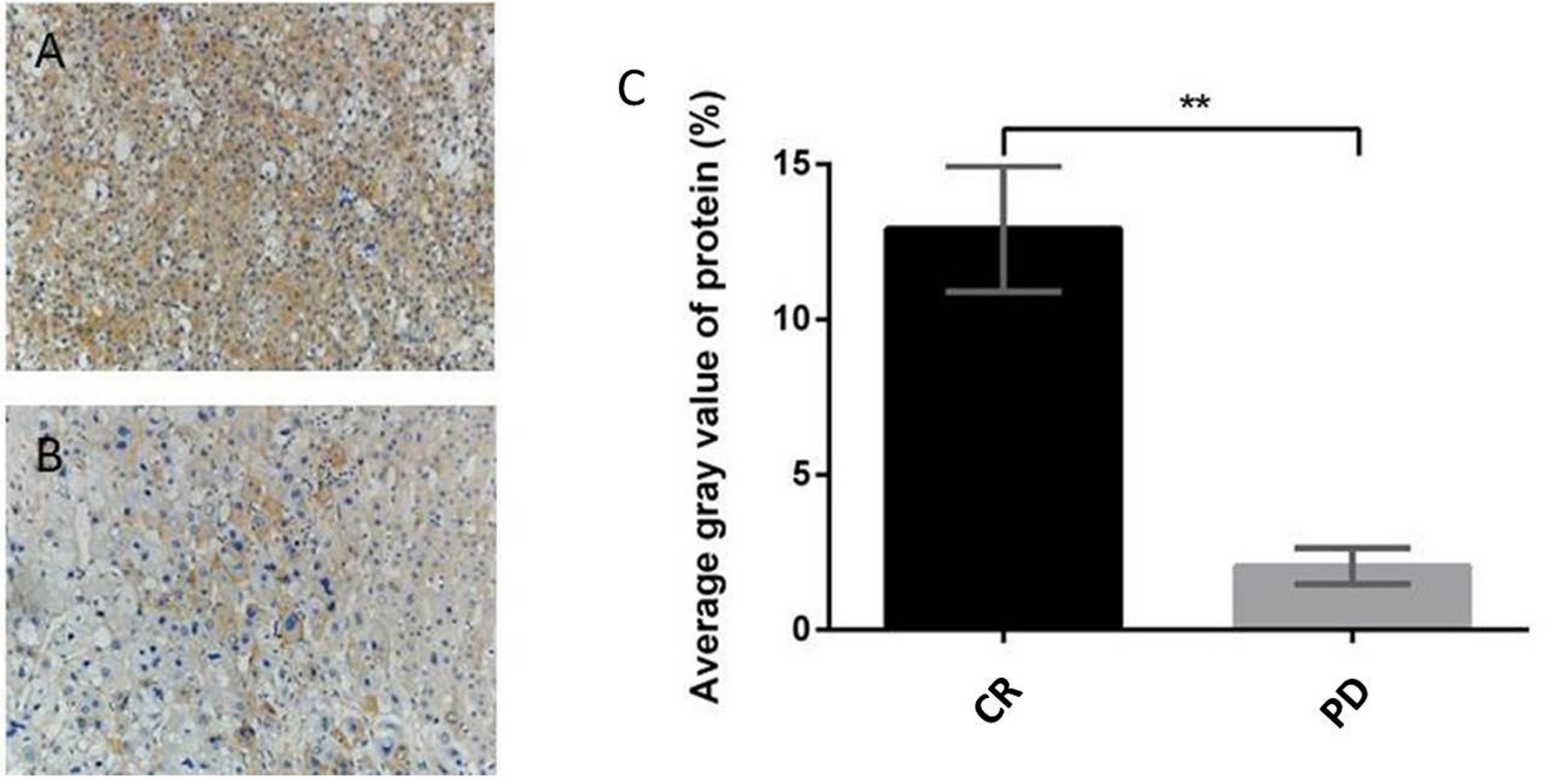
Immunohistochemical test of expression of CDKN2B/P15 protein in patients of VER+TACE therapy (**(A)** effective group, CR, ×200; **(B)** ineffective group, PD, ×200, **(C)** average gray value of protein). ^**^p<0.01 compared to the PD group.

The expression levels of CDKN2B/P15 protein in cancer tissue samples of VER responsive (CR, 12 cases) and unresponsive groups (PD, 10 cases) were tested using immunohistochemistry. The mean density (IOD/area) of the positive expression region was analyzed using Image-Pro Plus 6.0 (IPP) (Figure 5). CDKN2B/P15 protein was mainly expressed in the nucleus and cytoplasm of cancer cells. In cancer tissues, the IOD/area of P15 of the VER responsive group was significantly higher than that of the VER unresponsive group (^**^p<0.01).

### Changes in VER reversal of chemotherapy resistance after loss of expression or overexpression of CDKN2B

**Figure 6:**
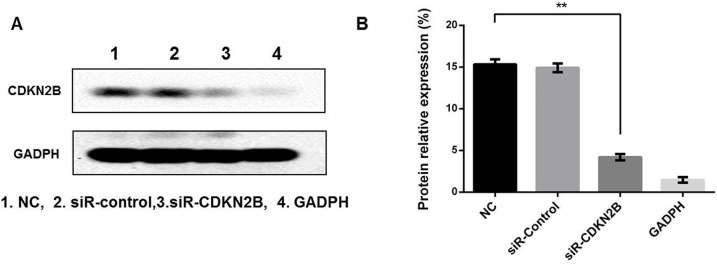
**(A)** Verification of loss of expression of CDKN2B genes in 7402 cells. **(B)** The profiles showed the protein gray value (%). ^**^p<0.01 compared to the NC group.

**Figure 7:**
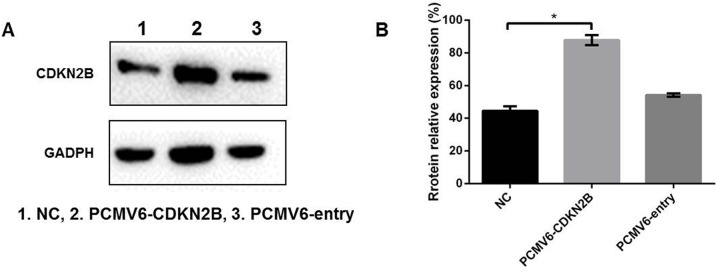
**(A)** Verification of overexpression of CDKN2B genes in 7721 genes. **(B)** The profiles showed the protein gray value (%). ^*^p<0.05 compared to the NC group.

**Figure 8:**
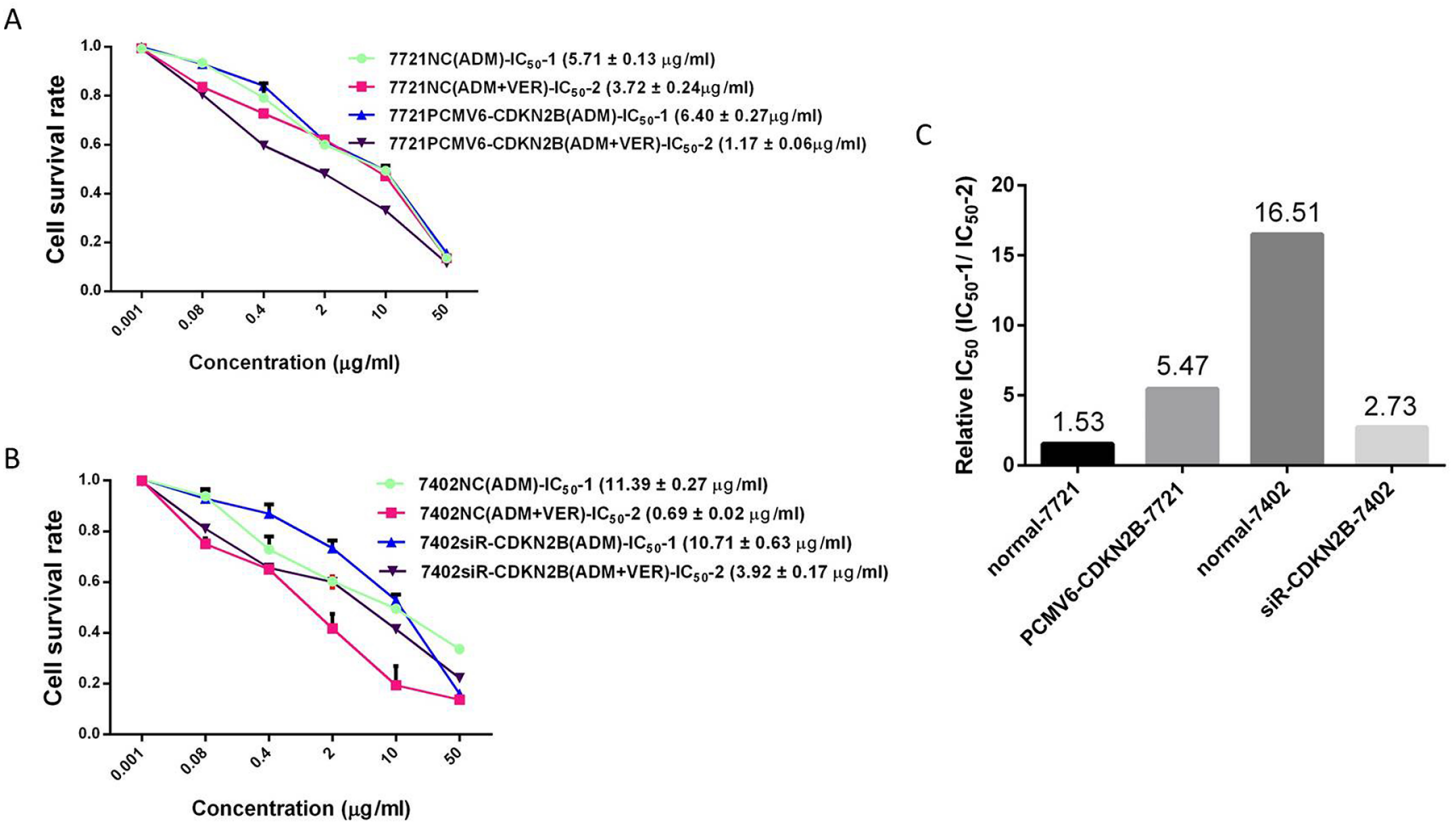
Changes of VER reversal of chemotherapy resistance after changing expression level of CDKN2B genes **(A)** Effect of different concentrations of ADM or ADM+VER on viability of 7721NC cells or 7721PCMV6+CDKN2B cells by MTT assay; **(B)** effect of different concentrations of ADM or ADM+VER on viability of 7402NC cells or 7402PCMV6+CDKN2B cells by MTT assay; **(C)** resistance evaluation to VER reversal ADM, (Relative IC_50_ = IC_50_-1/IC_50_-2). The IC_50_ values of four types of HCC cell lines, which were treated by chemotherapeutics in the absence (IC_50_-1) or in the presence (IC_50_-2) of VER.

The CDKN2B gene (siR-CDKN2B) was expressed in BEL-7402 cells and the transfection results were verified by western blot. As shown in Figure 6, CDKN2B protein expression in the control group was normal, and in the siR - CDKN2B group, the CDKN2B protein decreased normally, indicating the success of transfection. In the SMMC-7721 cells, the CDKN2B gene (PCMV6-CDKN2B) was overexpressed and the transfection results were verified by western blot, as shown in Figure 7: 1.NC, 3.PCMV6-entry group, CDKN2B protein had normal expression; in the CDKN2B group, CDKN2B protein expression was significantly increased. The CCK-8 method was used to determine the IC50 value of ADM and ADM + VER in HCC cells before or after the change of gene expression. When CDKN2B gene (siR-CDKN2B) and VER were removed in BEL-7402 cells, ADM chemotherapy resistance was significantly reduced. In the SMMC-7721 cells, overexpression of the CDKN2B gene (PCMV6-CDKN2B) significantly enhanced VER reversal of ADM chemotherapy resistance (Figure 8).

### Annexin V-PI double-staining test of HCC cell apoptosis

**Figure 9:**
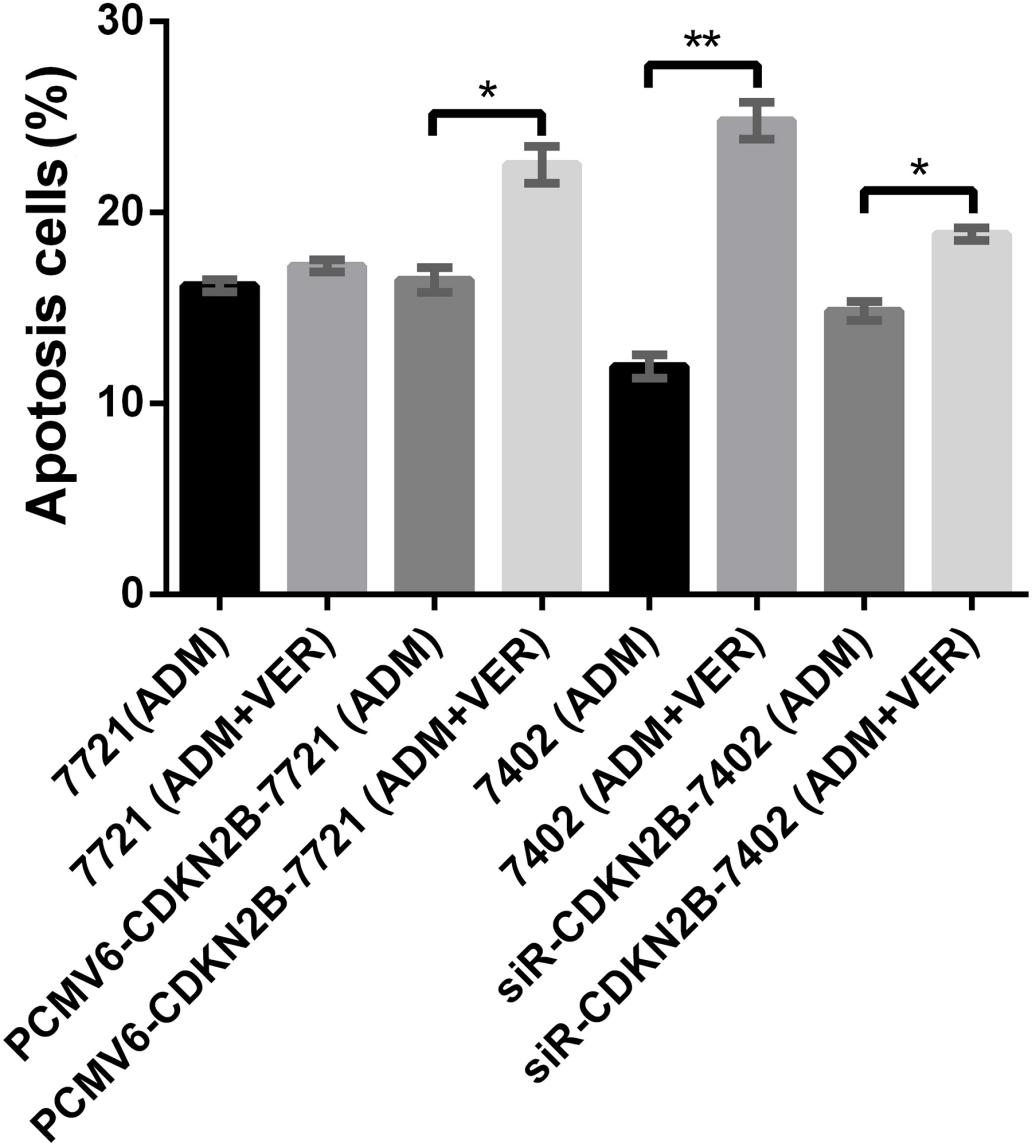
Promotion test of VER+ADM in cell apoptosis by flow cytometry

Figure 9 and Supplementary Figure 1 show that the cell apoptosis rates of SMMC- 7721 and BEL-7402 treated with ADM and ADM+VER when CDKN2B was overexpressed or silenced. The 7721 (ADM) and 7721 (ADM+VER) groups showed no significant difference in cell apoptosis at 16.4% and 17.2%, respectively. After overexpressing the CDKN2B gene (PCMV6-CDKN2B), the cell apoptosis rates of the 7721 (ADM) and 7721 (ADM+VER) groups changed to 17.1% and 22%, which represents a significant difference (^*^p<0.05. The results confirmed that VER obviously promotes the apoptosis of HCC cells in the presence of ADM which promotes overexpression of CDKN2B genes. The cell apoptosis rates of the 7402 (ADM) and 7402 (ADM+VER) groups were significantly different at 12.6% and 24.7%, respectively. After silencing CDKN2B gene (siR-CDKN2B) expression, the cell apoptosis rates of 7402 (ADM) and 7402 (ADM+VER) groups changed to 14.8% and 18.3%. The difference between the two groups was not significant compared with the cells before CDKN2B gene silencing.

The results revealed that silencing CDKN2B genes reduces the ability of ADM to kill HCC cells in the presence of VER. This result reflects that CDKN2B genes are critically involved in the promotion of cell apoptosis by ADM+VER. Upregulating the expression level of CDKN2B enhances ADM+VER induced cell apoptosis, whereas downregulating the expression level of CDKN2B weakens that promotion.

## DISCUSSION

VER is a calcium channel inhibitor. P-glycoprotein (P-gP), known as multidrug resistance protein 1 (MDR1), is viewed as the main target for VER reversal of tumor drug resistance [7]. As a transmembrane protein in the ATP binding cassette transporter protein family, P-gP can transfer drug from the inside of cells to the outside, thus enabling tumor cells to generate drug resistance. Research shows that VER can prevent the coupling of energy released by ATP hydrolysis and transport function of P-gP, causing loss of P-gP drainage capacity. VER may fight with drugs for P-gP, thereby reducing drug drainage to outside of cells [8]. Alternatively, VER combined with chemical drugs can inhibit P-gP expression [9]. However, no high expression of MDR1 is detected in Taxol (TAX)-resistant ovarian cancer cells and ADM-resistant gastric cancer cells [10, 11]. Chiu et al. [12] confirmed that VER reversal of multidrug resistance in lung carcinoma cells is unrelated to the expression of P-gP. No simple correlation between the expression levels of P-gP in four HCC cell lines and VER reversal of chemotherapy resistance was found. This result reveals that the reversal effect of VER is not directly related to the expression level of P-gP. All of these results suggest that the VER reversal of drug resistance in HCC cells might involve other targets, outside of P-gP.

To explore new targets that can mediate VER reversal of drug resistance in HCC cells, VER reversal of resistances to L-OHP, ADM, and 5-FU in four HCC cell lines (SMMC-7721, BEL-7402, HepG2, and QGY-7703) were evaluated in this study. We found that BEL-7402 had the strongest resistance to VER + ADM (Relative IC_50_=16.52), significantly stronger than SMMC-7721 (Relative IC_50_=1.53). On this basis, SMMC-7721 and BEL-7402 were used as the research objects to screen a group of genes that may mediate resistance to VER reversal of ADM resistance. According to qRT-PCR and Western blot verifications, upregulating the expression of CDKN2B/p15 can increase resistance to VER + ADM. The immunohistochemical test of expression levels of CDKN2B/p15 protein in VER effective/ineffective groups revealed that the expression level of CDKN2B/p15 protein in VER sensitive group was higher than the expression level of CDKN2B/p15 protein in the VER insensitive group.

To further confirm the role of CDKN2B in mediating the drug resistance of liver cancer, the expression of CDKN2B gene (siR-CDKN2B) was silenced in BEL-7402 cells and was overexpressed (PCMV6-CDKN2B) in SMMC-7721 cells. IC50 values of ADM and ADM + VER groups were detected by CCK-8 before or after gene expression. When CDKN2B gene (siR-CDKN2B) was silenced in BEL-7402 cells, and the resistance of VER to ADM chemotherapy was significantly decreased. In the SMMC-7721 cells, the CDKN2B gene (PCMV6-CDKN2B) was overexpressed and the resistance of VER to ADM chemotherapy was significantly enhanced. This result reveals that CDKN2B/P15 participates in the regulation of VER reversal of liver cancer chemotherapy resistance. Upregulating the expression of CDKN2B/p15 can enhance the resistance of VER reversal chemotherapy.

CDKN2B (cyclin dependent protein kinase inhibitor 2B, or called p15 and INK4B) belongs to the INK4 protein family. Also known as multiple tumor suppressor (MTS1), it can inhibit the activity of cyclin-dependent kinase 4 (CDK4) or CDK6, which thereby inhibit cell cycle and cause G1 retardation of cells, thus inhibiting tumor cell proliferation and facilitating tumor cell apoptosis [13, 14]. Some research has demonstrated that upregulating the expression of CDKN2B can promote the G1 retardation of HepG2/DDP, inhibit tumor cell proliferation, and enhance the accumulation and retention of chemotherapeutic drugs in cells [15]. Zu et al. found that the combined use of VER and ADM can facilitate the apoptosis of K562/P-gP (+) and K562/P-gP (-) in leukemia cells and that Caspase 3 is an important molecule that causes apoptosis [16]. Thus far, the participation of CDKN2B in the regulation of VER reversal of chemotherapy resistance has not been reported. In this study, we found that changes in the expression level of CDKN2B correlates with the promotion of cell apoptosis by VER+ADM. Upregulating the expression level of CDKN2B enhanced the promotion of cell apoptosis by VER+ADM, whereas downregulating the expression level of CDKN2B genes weakened such promotion. Future research will explore the functional mechanism, action signal transduction pathway, and signal molecules of CDKN2B genes.

## MATERIALS AND METHODS

### Materials

Oxaliplatin (L-OHP, Jiangsu Hengrui, 50mg/pc), doxorubicin hydrochloride (ADM, Zhejiang Haizheng, 10mg/pc), 5-fluorouracil (5-FU, Jiangsu Nantong Jinghua Pharmacy, 0.25g/10ml), and VER (Verapamil, VER, Shanghai Hefeng Pharmacy, 5mg/2ml) were supplied by our hospital. The CCK-8 kit was purchased from Japan Dojindo Laboratories. The RNA extraction and reverse transcription kit were purchased from TIANGEN. 2×SYBR Green universal qPCR Master Mix was purchased from TIANGEN. Mouse-anti human CDKN2B/P15 and P-gP primary antibody were purchased from Abcam. The GADPH antibody was purchased from Sigma. The second antibody labeled by sheep-anti mouse HRP was purchased from Guizhou Jinqiao Biotechnology. The high-flux sequencing was performed by Guangzhou Ruibo. siRNA for gene transfection was purchased from Guizhou Ruibo. The overexpression plasmid and empty carrier TrueORF GOLD type were purchased from ORIGENE. The annexin V-PI double-staining kit was purchased from Beijing Beibo. The Lipofectamine 3000 was purchased from Invitrogen. Primer design and synthesis were performed by Shanghai Shanjing Biotechnology Company.

### Cell culture

Cells were cultured normally in high-glucose DMEM culture solution containing 10% FCS under 37°C, 5% CO_2_, and saturation humidity. Human HCC cell lines in the logarithmic phase were treated using 0.25% trypsinization. The solution was changed every 24 h. When cells were in a monolayer dense distribution, the cell line was rinsed using PBS and then passed after being digested by 0.25% trypsin. The cells in the logarithmic phase were collected for the following experiment.

### IC_50_ value test of hepatoma carcinoma cells by CCK-8 method

Chemotherapeutics were diluted 1:4 or 1:5. The final concentrations of 5-FU were set as 200, 40, 8, 1.6, and 0.32 ug/mL. The final concentrations of ADM were set as 50, 10, 2, 0.4, and 0.08 ug/mL. The final concentrations of L-OHP were 100, 20, 4, 0.8, and 0.16 ug/mL. The final concentration of VER was 4.91 ug/mL.

HCC cells in the logarithmic phase were collected, digested by 0.25% trypsinization, and prepared into a single-cell suspension using high-glucose DMEM. The cells were inoculated into a piece of a 96-hole culture plate at a density of 2×10^5^ cells/mL (100 μL/hole), cultured in an incubator at 37°C in 5% CO_2_ for 12 h. Different concentrations of chemotherapeutics diluted using high-glucose DMEM were added. Each dose was administered in sets of three reproducing wells (control groups are PBS group and blank control group). After culturing for 72 h, CCK8 agents were added (10 μL/hole) and incubated for 1 h. The PBS group was used as the control group, and the OD_450_ values of every group were tested. The concentration–effect curves were drawn using drug concentration and the OD_450_ as the horizontal and vertical axes, respectively, based on which axis the median inhibitory concentration (IC_50_) was determined.

Each type of cell was treated by VER (fixed concentration=4.91 ug/mL) combined with chemotherapeutics. The IC_50_ values (IC_50_-2) of four types of HCC cell lines (SMMC-7721, BEL-7402, HepG2, and QGY-7703), which were treated by VER and chemotherapeutics, were tested. Three independent experiments were performed.

The drug resistance efficiency index (antagonizing drug-resistance index) of VER reversal of chemotherapeutic resistance was evaluated using IC_50_-1/IC_50_-2. A high IC_50_-1/IC_50_-2 implies a high antagonizing drug-resistance index.

### High-flux transcriptome sequencing based on Illumina sequencing platform

The high-flux sequencing was accomplished with one pair of hepatoma carcinoma cells (SMMC-7721 and BEL-7402) with the most significant difference in drug resistance of VER reversal ADM chemotherapy. Each type of cell was divided into four groups, namely, normal group (NC), VER group (VER), ADM group (ADM), and VER combined with ADM groups (ADM+VER). Considering the toxicity of chemotherapeutics and subsequent experiments, ADM dose was selected to be 1/5 of the IC_50_-1 of the cell line. In other words, the ADM concentration of the 7721 cell groups was 1.14 ug/mL (IC_50_-1=5.71 ug/mL), and the ADM concentration of the 7402 cell groups was 2.28 ug/mL (IC_50_-1=11.40 ug/mL). The VER concentration was 4.91 ug/mL.

Using the Illumina 2000 sequencing platform, the raw data is filtered through the serial sequence and the low quality sequence to get high quality data (clean reads). We used the Bowtie2 software to compare the clean reads to the human reference gene set (version hg38) and the RSEM software for quantitative calculation of gene expression. Based on the results of expression analysis, we used DESeq (version 1.28.0) software for differential gene analysis. The differentially expressed genes were selected by the difference of logarithm (log2 (Fold change) |> 1) and significance level (q-value <0.05). The results of the differential genes were visualized using the R-language pheatmap package.

### Real-time quantitative PCR test of expressions of CDKN2B and P-gP in HCC cells

#### Primer design

Primers were designed by Primer 5.0: CDKN2B genes (ID: 1030 180 bp) hCDKN2BF:5’-TCCCAACGGAGTCAACCG-3’and hCDKN2BR:5’-AGCACCACCAGCGTGTCC-3’. The internal reference primer (18SrRNA) was F:5’- GTAACCCGTTGAACCCCATT-3’ and R:5’- CCATCCAATCGGTAGTAGCG-3’.

#### RNA extraction and target gene test

The RNA extraction and the quantitative test were accomplished according to specification of the kit. Cells were grouped according to the transcriptome sequencing grouping shown in Section 1.4. After the dilution of the primer, its specificity and annealing temperature were optimized. Next, the reaction mixture was prepared according to the following reaction system: 2×SYBR Green universal qPCR Master Mix 12.5 μL and 1.5 μL upstream/downstream primers, respectively, cDNA 3 μL, and then double distilled water to the final volume of 25 μL is added. Corresponding volumes were prepared and added into the PCR plate (25 μL/well) based on the quantity of detection samples. The reaction mixture was delivered to the tube bottom through centrifugation. PCR was performed under the following reaction conditions: pre-denaturation at 95°C for 15 min, PCR reaction (denaturation at 95°C for 10 s and annealing/extension at 60°C for 32 s, a total of 40 cycles), and construction of solubility curve. Finally, the data were read directly from the real-time fluorescence quantification PCR instrument.

### Western blot test of expression of CDKN2B/P15 proteins in HCC cells

Cells were inoculated into a piece of 6 well plate (5.0×10^5^ cells/well), and cell grouping was performed similarly to the cell grouping in Section 1.5. Drug-treated cells were collected for 30 min pyrolysis by RIPA lysate (containing protease inhibitor PMSF). Later, these cells were centrifuged at low-temperature and supernatant was collected. Protein quantification was completed using bovine serum albumin (BSA) as the standard. Proteins were separated by sodium dodecyl sulfate polyacrylamide gel electrophoresis (SDS-PAGE) and were then transferred to PVDF film. These proteins were blocked by 5% skim milk powder for 1 h. A protein antibody (β-actin=1:2000, P15 protein=1:1000) was added. The proteins were incubated overnight under 4°C and then rinsed four times (10 min each) by phosphate buffer (PBST). Next, a secondary antibody (1:5000) was added for 2 h warm incubation and rinsed by PBST. Next, a developing agent was added in the dark and photos were taken.

### CDKN2B/P15 protein expression test in cancer tissue samples of HCC patents by immunohistochemical method

#### Clinical data and grouping

A total of 22 cases of primary HCC in middle and terminal stages were selected in our hospitals. Lesion samples were extracted using liver puncture technology, and all cases were diagnosed as primary HCC. Each patient received one intervention every month or a total of two to four interventions. The therapeutic effects of these interventions to the patients were evaluated into two groups, namely, 12 cases of VER effective group (high cure rate, CR) and 10 cases of VER ineffective group (progression of disease, PD). These two groups have no significant difference in gender and age. Both groups were signed by the *Informed Consent* for the VER+TACE treatment (Supplementary Figure 2 and 3).

### VER+TACE therapy method and evaluation standard of therapeutic effect

Arteria femoralis was punctured using Seldinger technology. After VER, doxorubicin, L-OHP, and 5-FU were injected through the celiac trunk artery or variant target blood vessel. Conduit was inserted into the right or left hepatic artery or variant target blood vessels selectively, followed by the embolism emulsification with iodinated oil. The steps for drug injection were as follows: VER 25mg, ADM 40–50 mg/ml, L-OHP 100–150 mg/ml, and 5-FU 1000 mg/ml. The materials and dose of embolism were selected and estimated according to radiography results. After completing embolization, arteriography was performed determine the blockage of the hepatic artery. The tube was removed after satisfactory embolization. Local package compression bandage was performed after no bleeding was observed with 10–20 min of hemostasis by compression at the puncture point.

Therapeutic evaluation of primary HCC treatment includes: complete response (CR): lump disappearing or shrinking by over 75%, uniform accumulation of iodipin, complete blockage of tumor vessels or only few tumor vessels or tumor staining left in tumor edges; partial response (PR): lump shrinking by approximately 30%–75%, non-uniform accumulation of iodipin, filling area of iodipin reaching higher than 1/2 of the lump area, significant reduction of tumor vessels; stable disease (SD): lump shrinking smaller than 30%, patched flocculent accumulation of iodipin, filling area of iodipin smaller than 1/2 of the lump, no significant reduction of tumor vessels; progression of disease (PD): lump expansion, scattered spot accumulation or non-evident accumulation of iodipin, accumulation area of iodipin smaller than 1/3 of the lump, significant growth of tumor vessels, and formation of new hepatic artery portal vein fistula or hepatic artery and vein fistula.

### Immunohistochemical method and result analysis

The paraffin-embedded HCC tissue was collected and sliced into 4 um continuous sheets for dewaxing and hydration. These tissues were boiled in a microwave for 15 min to recover antigen. The working concentration of the primary antibody was 1:100. The flowchart of immunohistochemical method is as follows: sodium citrate buffer solution is used to repair heat antigen, immunohistochemical staining by conventional SP method, DAB color developing, and redyeing with hematoxylin. Known positive slices of HCC were used as the positive control and PBS replaced the primary antibody as the negative control.

Positive expression contains claybank particles on cell membrane and/or cytoplasm, which was judged by semi-quantitative results. The density mean (IOD/area) of the positive expression region was analyzed by Image-Pro Plus 6.0(IPP). The mean and standard deviation of every picture of slices in the same experimental group were calculated. The significance of differences in density mean among different experimental groups was analyzed using the statistical method.

### Overexpression and silencing of CDKN2B in HCC cells

The overexpression plasmid (PCMV6-AC-GFP) and the empty carrier (PCMV6-Entry) of CDKN2B genes were the TrueORF GOLD mode of the ORIGENE Company. Specific siRNA interference plasmid was confirmed to have no significant homology with the human genome through the BLAST homology retrieval of three siRNA sequence pairs (si-h-CDKN2B_001, 002, and 003) of CDKN2B genes by using the software of Ruibo Company.

si-h-CDKN2B_001: positive-sense strand 5-CGGAGUCAACCGUUUCGGGUU-3; antisense strand 5-CCCGAAACGGUUGACUCCGUU-3.

si-h-CDKN2B_002: positive-sense strand 5-GGGAUAUUUAGGAGUGUGUTT-3; antisense strand 5-ACACACUCCUAAAUAUCCCTG-3.

si-h-CDKN2B_003: positive-sense strand 5-GGGAUAUUUAGGAGUGUGUTT-3; antisense strand 5-ACACACUCCUAAAUAUCCCTG-3.

Negative control NControl_05815: positive-sense strand 5-UUCUCCGAACGUGUCACGUTT-3; antisense strand 5-ACGUGACACGUUCGGAGAATT-3.

These siRNAs were synthesized by Guangzhou Ruibo Company and purified by PAGE.

### Cell transfection steps

①All 7402 cells were cultured at 37°C, 5% of CO_2_, and saturation humidity with high-glucose DMEM solution containing 10% fetal calf serum and antibiotics.

②One day before the transfection, cells in the logarithmic phase were digested by 0.25% tyrisin and suspended by high-glucose DMEM solution containing 10% fetal calf serum and no antibiotics. Next, cells were inoculated into 6-well (5.0×105 cells) or 96-well plates (1.0×104 cells). The transfection began until 80%–90% cell fusion.

③Cell transfection was conducted according to the manufacturer's instructions for Lipofectamine 3000. 5 μL (6-well plate) or 0.25 μL (96-well plate) Lipofectamine 3000 were added into 125 μL (6-well plate) and 6.25ul (96-well plate) Optim-MEM solution, respectively. The mixtures were mixed rapidly, and allowed to settle for 5 min.

④About 10 μL (6-well plate) or 0.5 μL (96-well plate) of plasmid were added into 125 μL (6-well plate) and 6.25 μL (96-well plate) Optim-MEM solution. The mixtures were mixed rapidly and allowed to settle for 5 min.

⑤Drop the mixture in Step ③ into the mixture in Step ④. The mixtures were mixed rapidly, and allowed to settle for 20 min.

⑥The mixture in Step ⑤ was added into a petri dish drop by drop. Care was taken not lift up cells.

⑦The mixture was cultured in an incubator at 37°C with 5% CO_2_ and saturating humidity for 5 h. The culture solution was changed to DMEM solution, containing serum, but no double antibody for another 72h culture. Finally, the mixture was analyzed.

### HCC cell apoptosis test using Annexin V-PI double-staining method

Cell grouping referred to transcriptome sequencing grouping. Cells were cultured using 10% FBS RMPI-1640 in a single well of a 6-well plate for 24 h until approximately 70% cell confluence was observed. Later, the cells were digested when the tyrosine and pancreatin actions were terminated by 10% FBS culture medium. The solution was centrifuged under 4°C for 5 min at the rate of 1000 rpm and then washed twice using pre-cooled PBS. Cells were re-suspended in 100 μL binding buffer and then 2 μL of Annexin V-FITC was be added. The mixture was placed on ice for 15 min in the dark, and 400 μL PBS was added. Each sample recieved 1 μL PI solution before being loaded to the flow cytometer. The mixture was expelled uniformly to test HCC cell apoptosis rapidly. Apoptosis state was observed under a fluorescence microscope. Three independent experiments were performed.

### Statistical analyses

Using the Illumina Hiseq 2000 sequencing platform, Bowtie2 software was used to clean reads from the reference gene set (version hg38). RSEM software was used for quantitative expression of gene expression and DESeq (version 1.28.0) software for differential gene analysis. Differentially expressed genes were selected by difference (| log2 (Fold change) |> 1) and significance levels (q-value <0.05) [17, 18]. VER-induced differences in drug resistance, the level of expression of targets, the level of cell apoptosis, and other variables were analyzed by one-way ANOVA or Mann-Whitney U test using Excel (Microsoft, Redmond, WA) or Prism (Prism 6.0, GraphPad Inc, La Jolla, CA, USA) and expressed as mean ± standard deviation (x ± S). Each experiment was repeated at least 3 times, and P <0.05 was considered statistically significant.

## SUPPLEMENTARY MATERIALS FIGURES


