# Supplementary material for: CDKN2B is critical for verapamil-mediated reversal of doxorubicin resistance in hepatocellular carcinoma
Source: Oncotarget. 2017 Oct 26;8(66):110052–63. doi: 10.18632/oncotarget.22123 (PMC5746364; doi:10.18632/oncotarget.22123)
Supplement: Supplementary file 1 [file oncotarget-08-110052-s001.pdf]

CDKN2B is critical for verapamil-mediated reversal of doxorubicin resistance in hepatocellular carcinoma

SUPPLEMENTARY MATERIALS

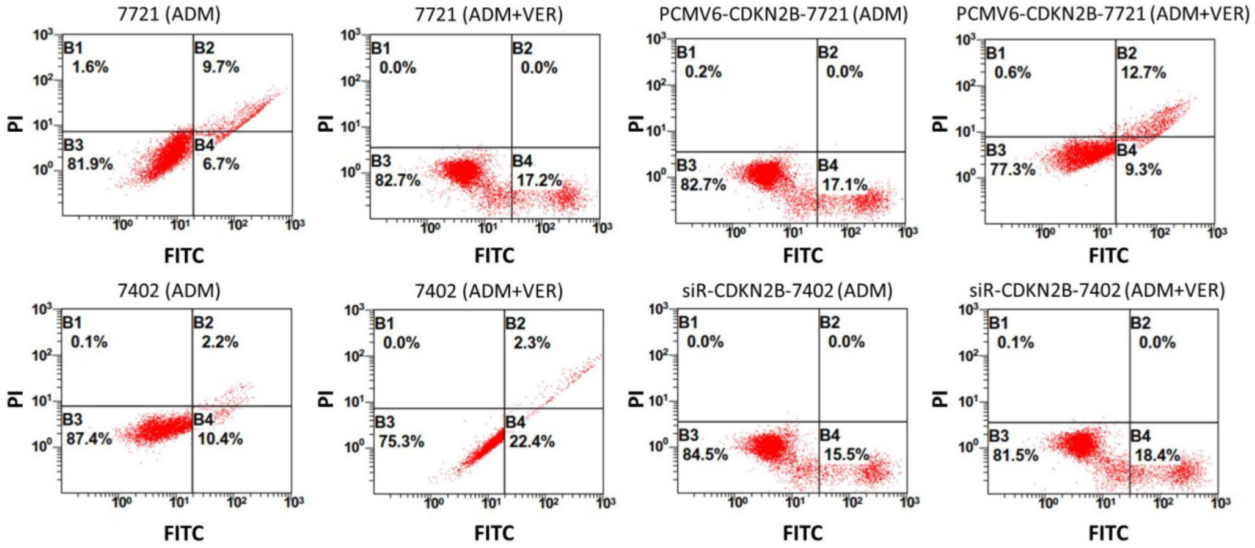

Supplementary Figure 1: Hepatoma carcinoma cell apoptosis rates before and after the use of VER+ADM.

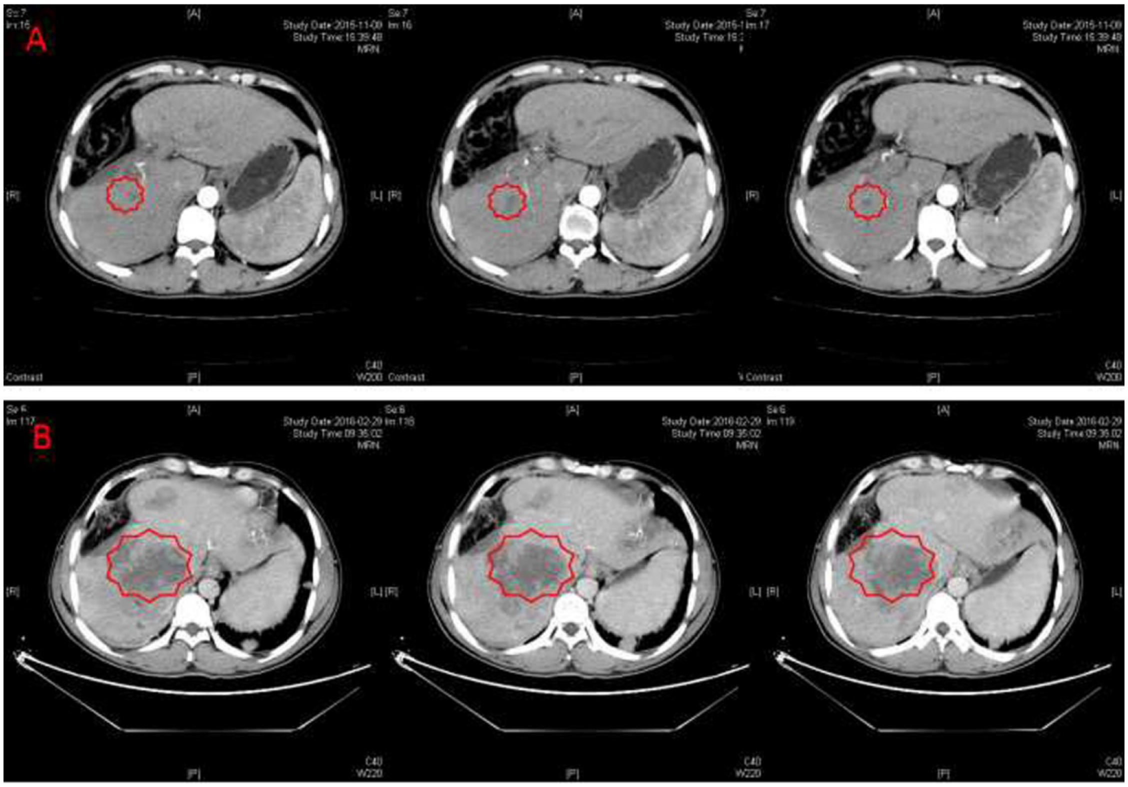

Supplementary Figure 2: Radiography of VER sensitive group (CR). **(A)** Before; **(B)** after.

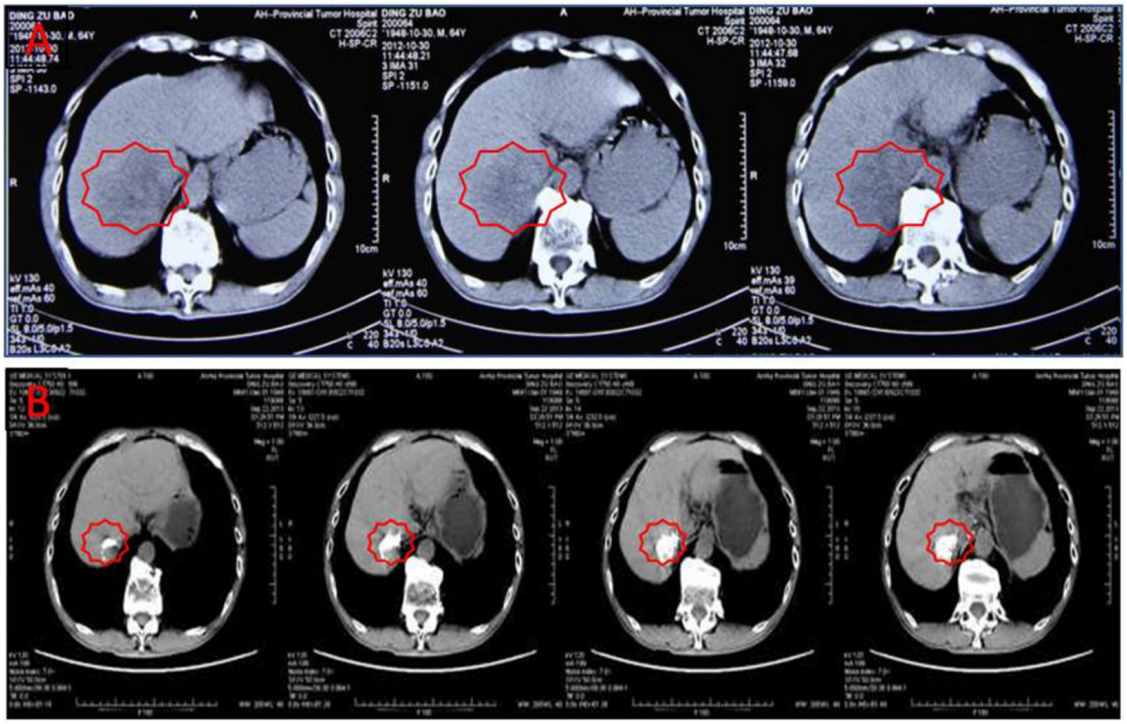

Supplementary Figure 3: Radiography of VER insensitive group (PD). **(A)** Before; **(B)** after.
